# Association of nonalcoholic fatty liver disease with new-onset atrial fibrillation stratified by age groups

**DOI:** 10.1186/s12933-024-02408-7

**Published:** 2024-09-12

**Authors:** Eun Ju Cho, Goh Eun Chung, Jeong-Ju Yoo, Yuri Cho, Kyu Na Lee, Dong Wook Shin, Yoon Jun Kim, Jung-Hwan Yoon, Kyungdo Han, Su Jong Yu

**Affiliations:** 1 Department of Internal Medicine and Liver Research Institute, Seoul National University College of Medicine, 101 Daehak-no, Jongno-gu, Seoul, 03080 Republic of Korea; 2 Department of Internal Medicine and Healthcare Research Institute, Seoul National University Hospital Healthcare System Gangnam Center, Seoul, Republic of Korea; 3 Department of Gastroenterology and Hepatology, Soonchunhyang University Bucheon Hospital, Bucheon-si, Gyeonggi-do Republic of Korea; 4 Center for Liver and Pancreatobiliary Cancer, National Cancer Center, Goyang, Republic of Korea; 5 Department of Biomedicine & Health Science, Catholic University, Seoul, Republic of Korea; 6 Department of Family Medicine, Supportive Care Center, Samsung Medical Center, Sungkyunkwan University School of Medicine, Seoul, Republic of Korea; 7 Department of Clinical Research Design and Evaluation, Department of Digital Health, Samsung Advanced Institute for Health Science, Seoul, Republic of Korea; 8 Department of Statistics and Actuarial Science, Soongsil University, Seoul, Republic of Korea

**Keywords:** Fatty liver index, Steatosis, Atrial fibrillation, Severity

## Abstract

**Background:**

The association between nonalcoholic fatty liver disease (NAFLD) and atrial fibrillation (AF) has been inconsistent, and the impact of hepatic fibrosis on this relationship remains uncertain. We investigated the association between NAFLD and the risk of new-onset AF across different age groups.

**Methods:**

A total of 3,179,582 participants from the 2009 Korean National Health Screening Program were divided into five groups based on NAFLD status: no NAFLD (fatty liver index [FLI] < 30); grade 1 NAFLD without advanced fibrosis (FLI 30–59 & BARD < 2); grade 1 NAFLD with advanced fibrosis (FLI 30–59 & BARD ≥ 2); grade 2 NAFLD without advanced fibrosis (FLI ≥ 60 & BARD < 2); and grade 2 NAFLD with advanced fibrosis (FLI ≥ 60 & BARD ≥ 2). The primary outcome was incident AF.

**Results:**

During the median follow-up of 9.3 years, 62,542 patients were diagnosed with new-onset AF. In the age- and sex-adjusted model, the risk of new-onset AF increased across NAFLD grades and fibrosis categories: grade 1 NAFLD without advanced fibrosis (hazard ratio [HR] 1.120, 95% confidence interval [CI]: 1.081–1.161); grade 1 NAFLD with advanced fibrosis (HR 1.275, 95% CI 1.251–1.300); grade 2 NAFLD without advanced fibrosis (HR 1.305, 95% CI: 1.252–1.360); and grade 2 NAFLD with advanced fibrosis (HR 1.627, 95% CI: 1.586–1.670). In the multivariate model, the excess risk of AF in patients with NAFLD and advanced fibrosis remained significant, even in participants aged 20–39 years.

**Conclusion:**

Patients with NAFLD had a higher risk of new-onset AF, which increased progressively with NAFLD severity, particularly in those aged 20–29 years.

**Supplementary Information:**

The online version contains supplementary material available at 10.1186/s12933-024-02408-7.

## Introduction

Nonalcoholic fatty liver disease (NAFLD) is a significant public health burden, affecting approximately 25% of the global population. About 30% of patients with NAFLD develop progressive steatohepatitis, which can advance to cirrhosis and hepatocellular carcinoma [1]. However, the leading cause of death in patients with NAFLD is cardiovascular disease (CVD) [2], with NAFLD identified as a risk factor for major adverse cardiovascular events (MACE), including incident heart failure and ischemic stroke [3, 4]. The burden of hepatic fibrosis is associated with an increased risk of MACE and all-cause mortality [5–8]. These findings suggest that NAFLD is a predictor of incident CVD and related outcomes.

Ultrasonography is the first-line diagnostic method for hepatic steatosis in clinical practice, but serum-based non-invasive tests are acceptable alternatives for large population-based epidemiological studies [9]. The fatty liver index (FLI) was developed as a simple and accurate marker of hepatic steatosis, derived from an algorithm using body mass index (BMI), waist circumference (WC), and levels of triglycerides (TG) and gamma-glutamyl transferase (GGT) [10]. The BARD score is a model to detect advanced liver fibrosis in patients with NAFLD, derived from a summation of the aspartate aminotransferase (AST)/alanine transaminase (ALT) ratio, BMI ≥ 28 kg/m^2^, and the presence of type 2 diabetes mellitus [11]. The FLI and BARD scores are frequently measured in clinical practice and can predict hepatic steatosis and fibrosis with acceptable performance in the general population [11–13].

The association between NAFLD and incident atrial fibrillation (AF) has been reported in recent years [14]. Previous meta-analyses have shown that patients with NAFLD are at an increased risk of AF compared with controls [15–17]. In addition, a nationwide population-based study found that the FLI positively correlated with incident AF risk for NAFLD [18, 19]. Currently, limited studies have considered the role of hepatic fibrosis in the association between AF and NAFLD. Moreover, research on the association between AF and NAFLD/fibrosis stratified by age is lacking.

Thus, we aimed to investigate the association between the risk of new-onset AF and NAFLD severity using the FLI and BARD scores across different age groups in a population-based, nationwide Korean cohort.

## Methods

### Data source

Data were obtained from the Korean National Health Insurance System (NHIS), a national insurer managed by the Korean government, which covers approximately 97% of the Korean population. The NHIS database contains various data items, including demographics, anthropometric measurements, laboratory tests, lifestyle behaviors, medical diagnostic codes based on the 10th revision of the International Classification of Diseases [ICD-10]), and treatment [20]. In this study, we used a customized NHIS database cohort comprising 40% of the Korean population, selected using stratified random sampling to ensure that the sample was representative of the entire population.

### Study population

**Fig. 1:**
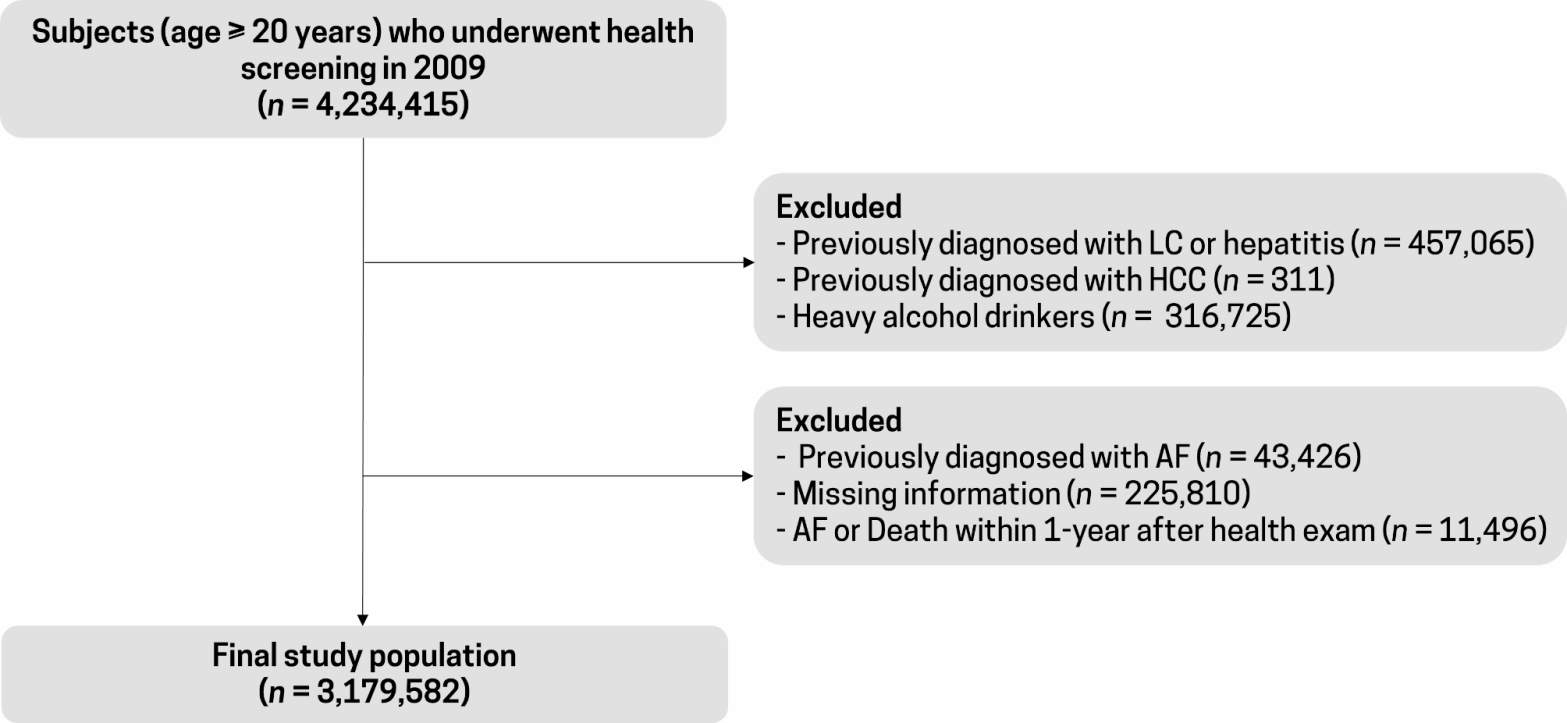
Flow chart of the study population

We enrolled 4,234,415 adults aged ≥ 20 years who underwent health screening examinations between January 1, 2009, and December 31, 2009. Among these, 1,043,337 participants were excluded based on the following criteria: (i) obvious liver disease such as liver cirrhosis (ICD-10 codes, K703 and K76) and hepatitis (ICD-10 codes, B15–B19) (*n* = 457,065), (ii) hepatocellular carcinoma (ICD-10 code, C22) (*n* = 311), (iii) excessive alcohol intake (> 30 g/day for men and > 20 g/day for women) (*n* = 316,725), (iv) prevalent AF (ICD-10-codes, I480–I484, I489) diagnosed before 2009 (*n* = 43,426), and (v) missing health examination data (*n* = 225,810). Additionally, we ascertained outcome events after a one-year lag to avoid reverse causality, which rendered an additional 11,496 individuals ineligible to participate. Finally, 3,179,582 participants were included in the study (Fig. 1).

The study protocol was approved by the Institutional Review Board (IRB) of Seoul National University Hospital (IRB no. 2401-130-1505). This study conformed to the ethical guidelines of the Declaration of Helsinki and its amendments. The requirement for informed consent was waived because the data used in this study were anonymized in accordance with confidentiality guidelines.

### Calculation of FLI and BARD

The FLI was calculated using the following formula [10].

$${\text{FLI = }}\frac{{\left[ {{\text{e}}^{{\left( \begin{subarray}{l} 0.{\text{953}} \times {\text{ln}}\left( {{\text{TG}}} \right) + 0.{\text{139}} \times {\text{BMI}} + 0.{\text{718}} \times {\text{ln}}\left( {{\text{GGT}}} \right) \\ + 0.0{\text{53}} \times {\text{WC}} - {\text{15}}.{\text{745}} \end{subarray} \right)}} } \right]}}{{\left[ {{\text{1 + e}}^{{\left( \begin{subarray}{l} 0.{\text{953}} \times {\text{ln}}\left( {{\text{TG}}} \right) + 0.{\text{139}} \times {\text{BMI}} + 0.{\text{718}} \times {\text{ln}}\left( {{\text{GGT}}} \right) \\ + 0.0{\text{53}} \times {\text{WC}} - {\text{15}}.{\text{745}} \end{subarray} \right)}} } \right]}} \times 100 $$

NAFLD was defined as an FLI ≥ 30 without obvious liver disease or excessive alcohol intake. The FLI has been validated in a Korean population with an acceptable area under the receiver operating characteristic curve [12, 21]. Among patients with NAFLD, advanced hepatic fibrosis was detected using the BARD score, which is derived from a summation of AST/ALT ratio ≥ 0.8 (two points), BMI ≥ 28 kg/m^2^ (one point), and type 2 diabetes mellitus (one point). A total score of 2–4 indicates advanced hepatic fibrosis [11].

We categorized the total population into five groups according to NAFLD status: no NAFLD (FLI < 30); grade 1 NAFLD without advanced fibrosis (FLI 30–59 & BARD < 2); grade 1 NAFLD with advanced fibrosis (FLI 30–59 & BARD ≥ 2); grade 2 NAFLD without advanced fibrosis (FLI ≥ 60 & BARD < 2); and grade 2 NAFLD with advanced fibrosis (FLI ≥ 60 & BARD ≥ 2). These indications were consistently applied across age groups and subpopulations.

### Outcome

The primary outcome was new-onset AF during the follow-up period, defined as at least one hospital visit with a diagnosis of AF (ICD-10 codes: I480–I484, I489) [14]. Participants were followed up until the occurrence of incident AF, deletion from the NHIS (death or immigration), or the end of the study period (December 31, 2019), whichever came first.

### Covariates

Health examination data, including height, weight, WC, and blood pressure, were obtained, and BMI was calculated by dividing weight (kg) by height (m^2^). Abdominal obesity was defined as WC ≥ 90 cm for males and ≥ 80 cm for females. Laboratory data included serum glucose, total cholesterol, triglycerides, high-density lipoprotein cholesterol, low-density lipoprotein (LDL) cholesterol, AST, ALT, and GGT levels.

Self-reported questionnaire data regarding smoking status, alcohol use, and physical activity were obtained. Smoking status was categorized as non-smoker, former smoker, or current smoker. Alcohol consumption was estimated by multiplying the amount of alcohol consumed per occasion with the frequency of alcohol intake per week. Participants were classified as non-drinkers or mild-to-moderate drinkers (alcohol intake: <30 g/day for males and < 20 g/day for females). Regular exercise was defined as high-intensity physical activity at least three times per week or moderate-intensity physical activity at least five times per week.

Comorbidities were defined using clinical data from the National Health Screening Program, ICD-10 codes, and prescription history, as follows: diabetes mellitus—use of hypoglycemic agents and insulin, ICD-10 codes (E11–14), or a fasting glucose level of ≥ 126 mg/dL; hypertension—history of antihypertensive medications, ICD-10 codes (I10–13 and I15), or elevated blood pressure (systolic blood pressure ≥ 140 mmHg or diastolic pressure ≥ 90 mmHg); dyslipidemia—ICD-10 code (E78), use of lipid-lowering medications, or a total cholesterol level > 240 mg/dL; and chronic kidney disease (CKD)—estimated glomerular filtration rate of ≤ 60 mL/min/1.73 m^2^ calculated using the Modification of Diet in Renal Disease formula.

### Statistical analysis

Baseline characteristics are expressed as percentages for categorical variables, mean ± standard deviation for normally distributed continuous variables, and geometric mean with 95% confidence interval (CI) for non-normally distributed continuous variables.

The incidence rate of new-onset AF (number of deaths per 1000 person-years) was also estimated. Cox proportional hazard analyses were performed to evaluate the association between FLI and the risk of new-onset AF.

Multivariable-adjusted models were used as follows: Model 1, adjusted for age and sex; and Model 2, adjusted for variables in Model 1, as well as income level, BMI, lifestyle habits (smoking status, alcohol consumption, and physical activity), and comorbidities (diabetes mellitus, hypertension, dyslipidemia, and CKD). Stratified analyses were performed according to sex, the presence of abdominal obesity (WC ≥ 90 cm for men and ≥ 80 cm for women), and the presence of diabetes. We tested the interactions between subgroups.

All statistical analyses were performed using SAS version 9.4 (SAS Institute, Cary, NC, USA). A two-tailed *p*-value of less than 0.05 was considered statistically significant.

## Results

### Baseline characteristics

The baseline characteristics of the study participants based on the presence or absence of new-onset AF are presented in Table 1. Patients with new-onset AF were older and more likely to be male, non-smokers, and non-drinkers. Traditional risk factors for CVD, including diabetes mellitus, hypertension, dyslipidemia, and CKD, were more prevalent in patients with new-onset AF than in those without new-onset AF. Higher BMI and WC, higher systolic and diastolic blood pressure, and higher levels of fasting glucose, total cholesterol, LDL cholesterol, and triglycerides were observed in patients with new-onset AF (all *P* < 0.001).

**Table 1:** Baseline characteristics of study population

Variables	AF (-)	AF (+)	*P* value
	(*n* = 3,117,040)	(*n* = 62,542)	
Age groups (years)			< 0.001
20–29	390,988 (12.5)	872 (1.4)	
30–39	614,350 (19.7)	2551 (4.1)	
40–49	839,306 (26.9)	6948 (11.1)	
50–59	653,142 (21.0)	13,026 (20.8)	
60–69	403,660 (13.0)	19,454 (31.1)	
≥ 70	215,594 (6.9)	19,691 (31.5)	
Sex			< 0.001
Male	1,604,765 (51.5)	34,688 (55.5)	
Female	1,512,275 (48.5)	27,854 (44.5)	
Income, Lowest Q1	621,968 (20.0)	12,514 (20.0	0.7326
Abdominal Obesity	571,023 (18.3)	20,739 (33.2)	< 0.001
Smoking			< 0.001
Non	1,961,743 (62.9)	39,584 (63.3)	
Ex	410,963 (13.2)	10,878 (17.4)	
Current	744,334 (23.9)	12,080 (19.3)	
Drinking			< 0.001
Non	1,730,268 (55.5)	40,611 (64.9)	
Mild	1,386,772 (44.5)	21,931 (35.1)	
Regular exercise	545,355 (17.5)	12,465 (20.0)	< 0.001
Diabetes mellitus	243,110 (7.8)	10,610 (17.0)	< 0.001
Hypertension	735,436 (23.6)	33,054 (52.9)	< 0.001
Dyslipidemia	539,974 (17.3)	16,955 (27.1)	< 0.001
Anti-diabetic drugs	156,787 (5.0)	391,811 (12.6)	< 0.001
Anti-hypertensive drugs	528,961 (17.0)	1,479,970 (47.5)	< 0.001
Anti-dyslipidamic drugs	271,805 (8.7)	588,497 (18.9)	< 0.001
CKD	210,007 (6.7)	8872 (14.2)	< 0.001
Age, years	46.7 ± 13.9	61.8 ± 12.5	< 0.001
BMI, kg/m^2^	23.6 ± 3.2	24.4 ± 3.3	< 0.001
Waist Circumference, cm	79.7 ± 9.1	83.8 ± 8.8	< 0.001
Systolic BP, mmHg	121.9 ± 15.0	128.5 ± 16.5	< 0.001
Diastolic BP, mmHg	76.0 ± 10.0	78.6 ± 10.5	< 0.001
Fasting glucose, mg/dL	96.5 ± 22.9	103.2 ± 29.8	< 0.001
Total cholesterol, mg/dL	195.3 ± 36.7	196.4 ± 38.3	< 0.001
HDL-C, mg/dL	56.0 ± 27.5	54.4 ± 31.0	< 0.001
LDL -C, mg/dL	114.4 ± 38.3	115.6 ± 39.5	< 0.001
Estimated GFR, mL/min/1.73m^2^	87.7 ± 45.6	81.4 ± 39.2	< 0.001
Triglyceride, mg/dL^a^	110 (109.9–110.1)	121.8 (121.2–122.3)	< 0.001

Values are presented as number (%) or mean ± standard deviation

*AF* atrial fibrillation, *BMI* body mass index, *WC* waist circumference, *BP* blood pressure, *HDL-C* high-density lipoprotein-cholesterol, *LDL-C* low-density lipoprotein-cholesterol, *CKD* chronic kidney disease, *GFR* glomerular filtration rate

^a^Geometric mean (95% confidence interval)

### Association between NAFLD severity and new-onset AF

During the median follow-up period of 9.3 years (interquartile range: 9.1–9.6), 62,542 (2.0%) patients were diagnosed with new-onset AF. In the age- and sex-adjusted model, patients with NAFLD had an increased risk of new-onset AF compared with those without the condition (grade 1 NAFLD without advanced fibrosis, hazard ratio [HR] = 1.120, 95% CI 1.081–1.161; grade 1 NAFLD with advanced fibrosis, HR = 1.275, 95% CI 1.251–1.300; grade 2 NAFLD without advanced fibrosis, HR = 1.305, 95% CI 1.252–1.360; grade 2 NAFLD with advanced fibrosis, HR = 1.627, 95% CI 1.586–1.670; Table 2). After adjusting for income level, BMI, smoking, alcohol consumption, physical activity, diabetes mellitus, hypertension, dyslipidemia, and CKD, the increased risk of new-onset AF in patients with NAFLD with advanced hepatic fibrosis remained significant (grade 1 NAFLD with advanced fibrosis, HR = 1.086, 95% CI 1.063–1.111; grade 2 NAFLD with advanced fibrosis, HR = 1.218, 95% CI 1.179–1.259, Table 2).

**Table 2:** The association between NAFLD grade and fibrosis categories with new-onset atrial fibrillation

Groups	No. of participants	No. of event	IR, per 1000 PY	HR (95% C.I)	
				Age and sex adjusted	Multivariable
No NAFLD	2,104,222	33,641	1.75	1 (Ref.)	1 (Ref.)
Grade 1 NAFLD without advanced fibrosis	204,666	3336	1.78	1.120 (1.081, 1.161)	0.957 (0.921, 0.993)
Grade 1 NAFLD with advanced fibrosis	510,080	15,812	3.43	1.275 (1.251, 1.300)	1.086 (1.063, 1.111)
Grade 2 NAFLD without advanced fibrosis	163,102	2516	1.68	1.305 (1.252, 1.360)	1.003 (0.959, 1.050)
Grade 2 NAFLD with advanced fibrosis	197,512	7237	4.09	1.627 (1.586, 1.670)	1.218 (1.179, 1.259)

Multivariable model was adjusted for age, sex, income levels, body mass index, lifestyle factors (smoking status, alcohol consumption, physical activity), diabetes mellitus hypertension, dyslipidemia and chronic kidney disease

*IR* incidence rate, *HR* hazard ratio, *PY* person years, *NAFLD* nonalcoholic fatty liver disease

### Risk of new-onset AF according to different age groups

**Fig. 2:**
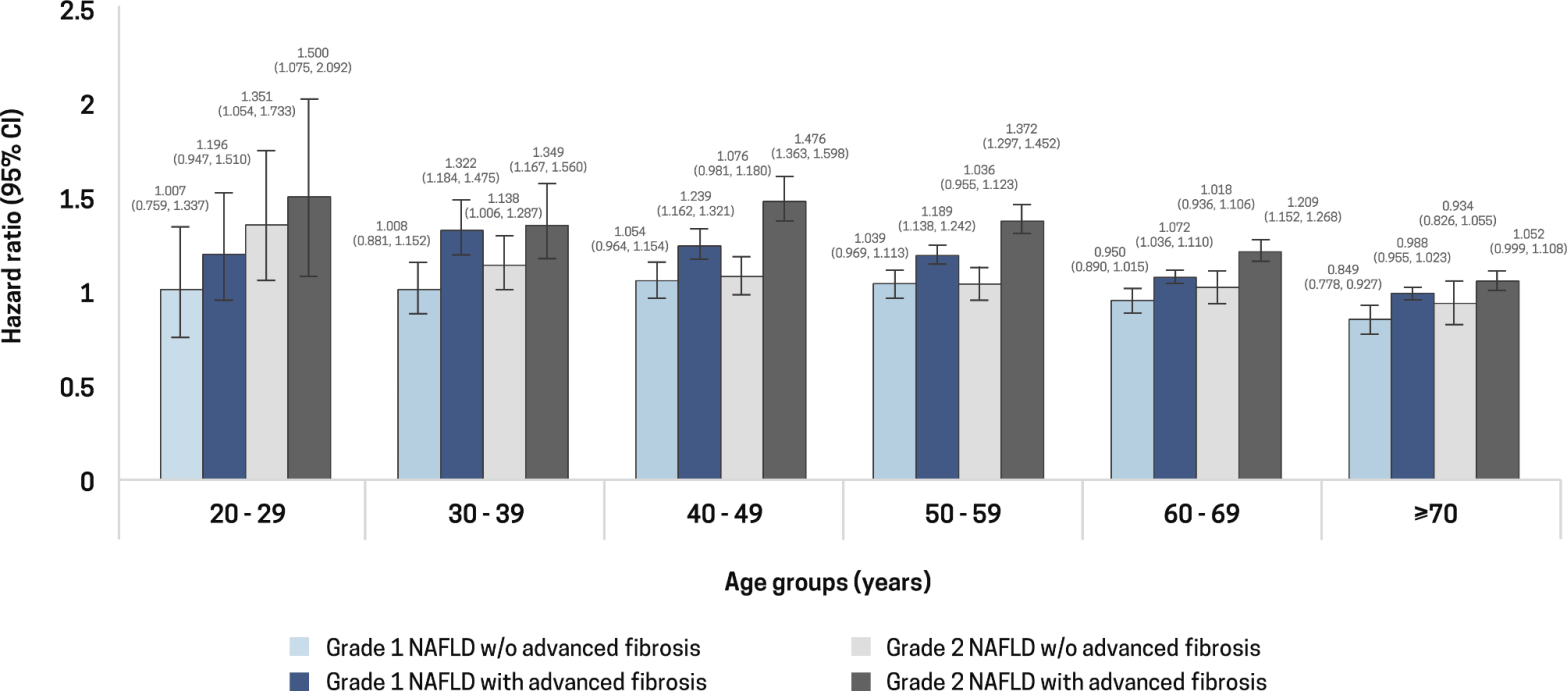
The association of nonalcoholic fatty liver disease severity with new-onset atrial fibrillation stratified by age groups Adjusted for age, sex, income levels, body mass index, lifestyle factors (smoking status, alcohol consumption, physical activity), diabetes mellitus hypertension, dyslipidemia, and chronic kidney disease. *BMI* body mass index, *CI* confidence interval

To account for the potential confounding effect of age, we stratified the participants into five groups: 20–29, 30–39, 40–49, 50–59, 60–69, and ≥ 70 years. The incidence of AF increased with age. In the age- and sex-adjusted model, the risk of new-onset AF was higher in participants with NAFLD than in those without NAFLD among participants aged ≥ 30 years, except those aged ≥ 70 years (Supplementary Table 1). In the multivariable analysis, the increased risk of new-onset AF remained significant among patients with NAFLD and advanced hepatic fibrosis aged ≥ 30 years (Fig. 2).

### Stratified analysis

**Fig. 3:**
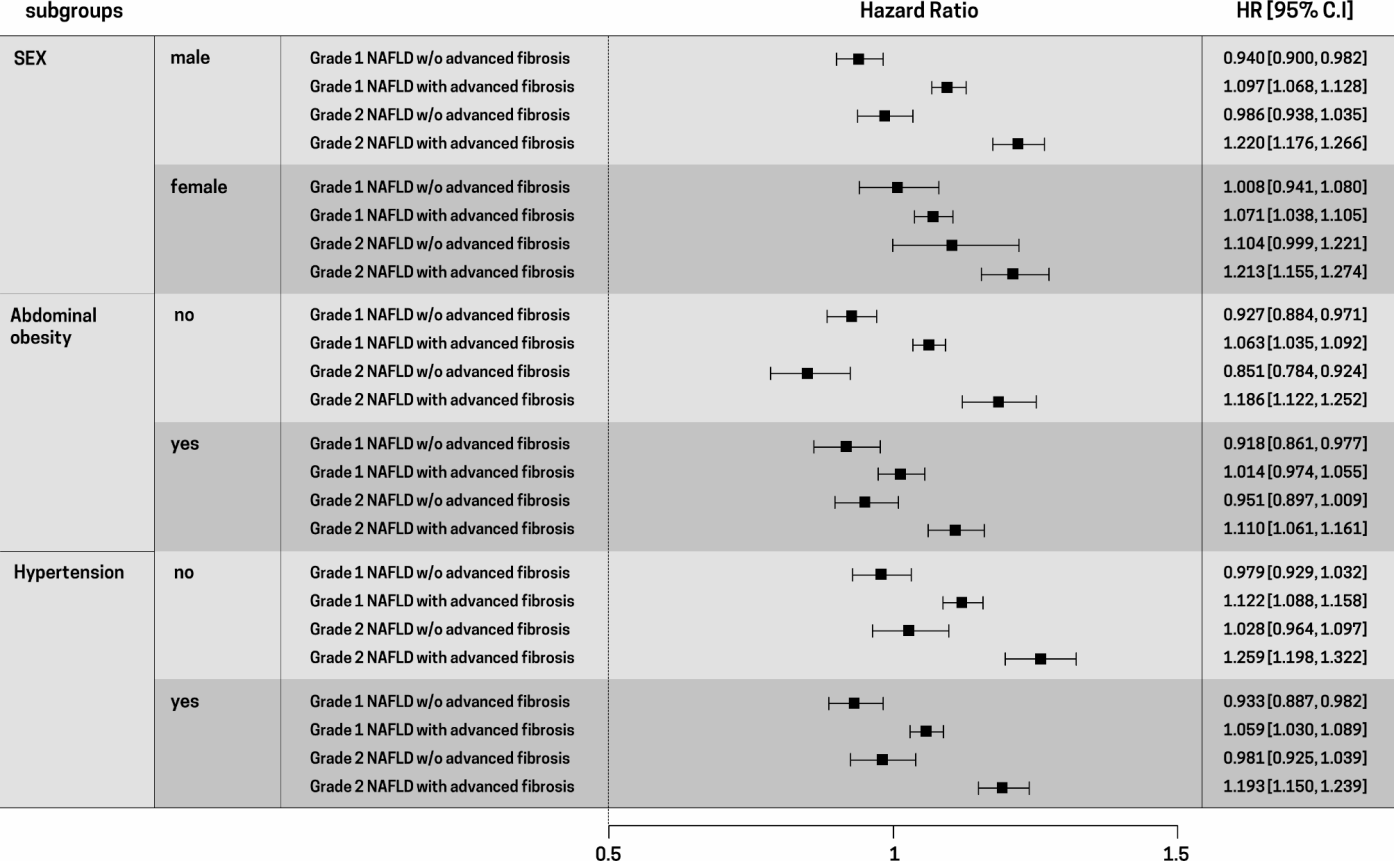
Stratified analysis by various groups Adjusted for age, sex, income levels, body mass index, lifestyle factors (smoking status, alcohol consumption, physical activity), diabetes mellitus hypertension, dyslipidemia, and chronic kidney disease. *BMI* body mass index, *HR* hazard ratio, *CI* confidence interval

In the stratified analyses by sex, the risk of new-onset AF in patients with grades 1 and 2 NAFLD with advanced fibrosis was more prominent in males (HR = 1.097 and 1.220, respectively) than in females (HR = 1.070 and 1.213, respectively; *P* for interaction = 0.042, Fig. 3).

Next, we stratified the participants by the presence of abdominal obesity (WC ≥ 90 cm for men and ≥ 80 cm for women). The increased risk of new-onset AF in patients with grades 1 and 2 NAFLD with advanced fibrosis was more prominent in those without abdominal obesity (HR = 1.063 and 1.186, respectively) than in those with abdominal obesity (HR = 1.014 and 1.110, respectively; *P* for interaction = 0.006, Supplementary Table 2).

In the subgroup analysis according to the presence of hypertension, the results were generally consistent with the main findings. The increased risk of new-onset AF in patients with grades 1 and 2 NAFLD with advanced fibrosis was more prominent in participants without hypertension (HR = 1.122 and 1.259, respectively) than in those with hypertension (HR = 1.059 and 1.193, respectively; *P* for interaction = 0.026, Supplementary Table 2).

## Discussion

In this nationwide population-based cohort study, the risk of new-onset AF increased in patients with NAFLD and advanced hepatic fibrosis, remaining significant even in young patients aged 20–39 years. The higher risk of AF in individuals with NAFLD and fibrosis is consistent with another study, indicating that NAFLD is associated with an enhanced risk of CVD [22].

The relationship between hepatic steatosis and AF in the general population has been established previously. A systematic review of 13 cohort studies linked NAFLD to a slight increase in AF risk (HR = 1.18, 95% CI 1.12–1.23) [23]. NAFLD predisposes patients to AF, regardless of the known risk factors for atherosclerosis [18]. NAFLD can significantly elevate the risk of AF in young adults owing to a complex interplay of biological and environmental factors. Biological factors, including metabolic syndrome, inflammation, oxidative stress, and autonomic dysfunction in NAFLD, as well as environmental factors such as diet, exercise, and lifestyle, contribute to the risk of AF [24–28]. Several studies have reported a significant association between metabolic dysfunction-associated fatty liver disease (MAFLD)/metabolic dysfunction-associated steatotic liver disease (MASLD) and AF. A recent study by Ohno et al. reported that patients with MAFLD were associated with a greater risk of developing AF (HR 1.51, 95% CI 1.46–1.57) [29]. In addition, patients with MASLD had significantly higher rates of incident AF (aHR = 1.26, 95% CI 1.18–1.35) [30]. Taken together, it is thought that there may be a significant relationship between MAFLD/MASLD and incident AF.

Assessing hepatic fibrosis in NAFLD can predict adverse clinical outcomes, including overall, cardiovascular, and liver-related mortality [31, 32]. Patients with NAFLD and advanced hepatic fibrosis were reported to have a higher AF risk [33, 34]. A retrospective cross-sectional study investigated the relationship between AF and advanced liver fibrosis among 6293 patients with NAFLD using non-invasive scoring systems: the NAFLD fibrosis score and Fibrosis-4. Consistent with our results, AF was independently associated with advanced liver fibrosis [34]. However, the study was limited by its single-center setting based on health examinations. In the present study, advanced hepatic fibrosis, measured using the BARD score, was associated with new-onset AF, which is consistent with previous reports. In addition, an increased risk of new-onset AF was observed in both grade 1 NAFLD with advanced fibrosis and grade 2 NAFLD with advanced fibrosis groups. These results suggest that, even in patients with mild steatosis, advanced hepatic fibrosis may be associated with an increased risk of AF. Although the exact mechanism underlying the association between AF and hepatic fibrosis is unclear, morphological and functional cardiac alterations are more pronounced in patients with severe fibrosis [35]. Moreover, cirrhosis significantly impairs cardiac function via vasodilation and hyperdynamic circulation [36]. Recently, Decoin et al. found that atrial fibrosis, a key factor in the occurrence of AF, was particularly increased in patients with MAFLD and a high liver fibrosis score, suggesting a liver-heart connection [37].

NAFLD influences the risk of AF not only in the general population [18] but also in younger age groups (< 30 years) [19]. In a recent systematic review, individuals aged < 40 years with NAFLD were at high risk of AF (HR = 2.00, 95% CI 1.12–3.57, *P* = 0.02) [38]. Similarly, Rho et al. reported an association between FLI and AF that was significant only in patients aged < 65 years [18]. Thus, we hypothesized a significant interaction between NAFLD severity and the risk of AF across different age groups. The risk of new-onset AF increased with an increase in NAFLD severity in all age groups. These findings suggest an independent association between NAFLD and AF regardless of age, except for patients aged ≥ 70 years. In addition, the association between NAFLD severity and the risk of new-onset AF was highest in participants aged 20–29 years, particularly those with grade 2 NAFLD with advanced fibrosis. These results indicate the need to closely monitor and manage NAFLD in young adults to mitigate the risk of AF.

The sex-stratified analysis showed that the association of new-onset AF in patients with grade 2 NAFLD and advanced fibrosis was slightly more pronounced in men than in women. In contrast, a previous study in Korea reported a more pronounced association between AF and NAFLD in women [18]. These discrepant results could be due to the heterogeneity in the FLI cutoff criteria and the use of BARD scores in our study.

Further, to evaluate the effect of central obesity and hypertension, we performed a stratified analysis based on the presence or absence of abdominal obesity or hypertension. The risk of new-onset AF in patients with grade 2 NAFLD and advanced fibrosis was more pronounced in those without abdominal obesity or hypertension compared to those with these conditions. This finding indicates a stronger association between new-onset AF and NAFLD severity in individuals without abdominal obesity or hypertension. These results are consistent with a previous study showing an increased risk of CVD in patients with grades 1 and 2 NAFLD without abdominal obesity or hypertension [22]. The mechanism underlying this interaction remains to be determined and warrants further investigation in future studies.

This study has several limitations. First, although diagnosing hepatic steatosis typically requires imaging or biopsy, these methods are expensive, invasive, and impractical for large population-based cohorts. Alternatively, the FLI has been used to define hepatic steatosis in several large-population studies utilizing claims data [22, 39]. However, the FLI has limited accuracy in quantifying hepatic steatosis and differentiating simple steatosis from steatohepatitis [40]. Additionally, the BARD score has high false positivity and is less specific compared to other biomarkers, such as the NAFLD fibrosis or Fibrosis-4 scores [41]. Despite these limitations, the BARD score has been used in previous studies owing to data availability constraints [42, 43]. Second, unmeasured confounders, such as inflammation or insulin resistance, could have influenced the results [25, 44]. Nevertheless, our findings remained robust after multivariate adjustment for conventional cardiovascular risk factors. Third, due to the cross-sectional design of this study, we could not establish a cause-and-effect relationship between NAFLD severity and new-onset AF. In addition, interpreting age-stratified results requires careful consideration of potential time bias and confounding variables. Future research with longitudinal study designs is needed to enhance our understanding in this area. Finally, the study population was comprised of participants from a single country, which may introduce selection bias. Because of differences in lifestyle habits, cultural/genetic backgrounds and body composition across geographic or ethnic populations, the results of this study may not be generalizable to other ethnic groups. Further validation studies across different ethnic groups are required to across different ethnic groups.

In conclusion, NAFLD severity, assessed using the FLI and BARD scores, was significantly associated with the risk of new-onset AF, suggesting that NAFLD severity may serve as a prognostic factor for new-onset AF, particularly in patients aged 20–29 years. Clinicians should be aware of the association between advanced NAFLD and AF in young adults, as timely identification of these patients may enable early intervention to prevent and treat AF.

## Electronic supplementary material

Supplementary Material 1

## Data Availability

We used the claim data provided by the Korean National Health Insurance Service (NHIS) database. Data can only be accessed by visiting the NHIS datacenter, after approval from data access committee of NHIS (https://nhiss.nhis.or.kr/bd/ab/bdaba001cv.do).
