# Supplementary material for: Association of nonalcoholic fatty liver disease with new-onset atrial fibrillation stratified by age groups
Source: Cardiovasc Diabetol. 2024 Sep 12;23:340. doi: 10.1186/s12933-024-02408-7 (PMC11395983; doi:10.1186/s12933-024-02408-7)
Supplement: Supplementary file 1 — Supplementary Material 1 [file 12933_2024_2408_MOESM1_ESM.docx]

**Supplementary Table 1.** The association between NAFLD grade and fibrosis categories with new-onset atrial fibrillation stratified by age groups.

|  |  | No. of participants | No. of event | IR, per 1000 PY | HR (95% C.I) | |
| --- | --- | --- | --- | --- | --- | --- |
| Age groups | Groups |  |  |  | Age and sex adjusted | Multivariable |
| 20 - 29 years | No NAFLD | 328,482 | 633 | 0.21 | 1 (Ref.) | 1 (Ref.) |
|  | Grade 1 NAFLD without advanced fibrosis | 17,436 | 52 | 0.32 | 1.203 (0.907, 1.597) | 1.007 (0.759, 1.337) |
|  | Grade 1 NAFLD with advanced fibrosis | 23,544 | 80 | 0.37 | 1.429 (1.132, 1.803) | 1.196 (0.947, 1.510) |
|  | Grade 2 NAFLD without advanced fibrosis | 14,929 | 70 | 0.51 | 1.887 (1.474, 2.416) | 1.351 (1.054, 1.733) |
|  | Grade 2 NAFLD with advanced fibrosis | 7,469 | 37 | 0.54 | 2.092 (1.501, 2.914) | 1.500 (1.075, 2.092) |
| 30 - 39 years | No NAFLD | 399,214 | 1,321 | 0.36 | 1 (Ref.) | 1 (Ref.) |
|  | Grade 1 NAFLD without advanced fibrosis | 57,448 | 258 | 0.48 | 1.168 (1.022, 1.335) | 1.008 (0.881, 1.152) |
|  | Grade 1 NAFLD with advanced fibrosis | 72,745 | 425 | 0.63 | 1.525 (1.367, 1.701) | 1.322 (1.184, 1.475) |
|  | Grade 2 NAFLD without advanced fibrosis | 56,615 | 329 | 0.63 | 1.504 (1.333, 1.697) | 1.138 (1.006, 1.287) |
|  | Grade 2 NAFLD with advanced fibrosis | 30,879 | 218 | 0.77 | 1.814 (1.572, 2.094) | 1.349 (1.167, 1.560) |
| 40 - 49 years | No NAFLD | 569,171 | 3,712 | 0.70 | 1 (Ref.) | 1 (Ref.) |
|  | Grade 1 NAFLD without advanced fibrosis | 58,413 | 562 | 1.04 | 1.232 (1.127, 1.347) | 1.054 (0.964, 1.154) |
|  | Grade 1 NAFLD with advanced fibrosis | 118,798 | 1,329 | 1.21 | 1.451 (1.362, 1.545) | 1.239 (1.162, 1.321) |
|  | Grade 2 NAFLD without advanced fibrosis | 49,226 | 545 | 1.20 | 1.408 (1.287, 1.542) | 1.076 (0.981, 1.180) |
|  | Grade 2 NAFLD with advanced fibrosis | 50,646 | 800 | 1.72 | 1.995 (1.848, 2.154) | 1.476 (1.363, 1.598) |
| 50 - 59 years | No NAFLD | 412,002 | 6,478 | 1.70 | 1 (Ref.) | 1 (Ref.) |
|  | Grade 1 NAFLD without advanced fibrosis | 43,145 | 933 | 2.35 | 1.212 (1.131, 1.298) | 1.039 (0.969, 1.113) |
|  | Grade 1 NAFLD with advanced fibrosis | 130,534 | 3,191 | 2.67 | 1.384 (1.326, 1.444) | 1.189 (1.138, 1.242) |
|  | Grade 2 NAFLD without advanced fibrosis | 28,195 | 688 | 2.67 | 1.334 (1.232, 1.443) | 1.036 (0.955, 1.123) |
|  | Grade 2 NAFLD with advanced fibrosis | 52,292 | 1,736 | 3.67 | 1.828 (1.733, 1.928) | 1.372 (1.297, 1.452) |
| 60 - 69 years | No NAFLD | 247,846 | 9,913 | 4.44 | 1 (Ref.) | 1 (Ref.) |
|  | Grade 1 NAFLD without advanced fibrosis | 21,352 | 993 | 5.18 | 1.104 (1.034, 1.178) | 0.950 (0.890, 1.015) |
|  | Grade 1 NAFLD with advanced fibrosis | 105,384 | 5,460 | 5.80 | 1.246 (1.205, 1.288) | 1.072 (1.036, 1.110) |
|  | Grade 2 NAFLD without advanced fibrosis | 11,224 | 616 | 6.18 | 1.285 (1.184, 1.394) | 1.018 (0.936, 1.106) |
|  | Grade 2 NAFLD with advanced fibrosis | 37,308 | 2,472 | 7.57 | 1.586 (1.518, 1.658) | 1.209 (1.152, 1.268) |
| ≥70 years | No NAFLD | 147,507 | 11,584 | 9.82 | 1 (Ref.) | 1 (Ref.) |
|  | Grade 1 NAFLD without advanced fibrosis | 6,872 | 538 | 9.49 | 0.991 (0.909, 1.080) | 0.849 (0.778, 0.927) |
|  | Grade 1 NAFLD with advanced fibrosis | 59,075 | 5,327 | 11.08 | 1.158 (1.121, 1.196) | 0.988 (0.955, 1.023) |
|  | Grade 2 NAFLD without advanced fibrosis | 2,913 | 268 | 11.40 | 1.182 (1.047, 1.334) | 0.934 (0.826, 1.055) |
|  | Grade 2 NAFLD with advanced fibrosis | 18,918 | 1,974 | 13.12 | 1.382 (1.317, 1.449) | 1.052 (0.999, 1.108) |

IR, incidence rate; HR, hazard ratio; PY, person years; NAFLD, nonalcoholic fatty liver disease

Multivariable model was adjusted for age, sex, income levels, body mass index, lifestyle factors (smoking status, alcohol consumption, physical activity), diabetes mellitus hypertension, dyslipidemia and chronic kidney disease.

**Supplementary Table 2.** Stratified analysis

|  |  | Groups | N | Event | IR | HR (95% C.I) | P for interaction |
| --- | --- | --- | --- | --- | --- | --- | --- |
| Sex | Male | No NAFLD | 852,907 | 15,807 | 2.04 | 1 (Ref.) | 0.042 |
|  |  | Grade 1 NAFLD without advanced fibrosis | 166,450 | 2,471 | 1.62 | 0.940 (0.900, 0.982) |  |
|  |  | Grade 1 NAFLD with advanced fibrosis | 32,7270 | 9,423 | 3.20 | 1.097 (1.068, 1.128) |  |
|  |  | Grade 2 NAFLD without advanced fibrosis | 146,829 | 2,111 | 1.57 | 0.986 (0.938, 1.035) |  |
|  |  | Grade 2 NAFLD with advanced fibrosis | 145,997 | 4,876 | 3.73 | 1.220 (1.176, 1.266) |  |
|  | Female | No NAFLD | 1,251,315 | 17,834 | 1.55 | 1 (Ref.) |  |
|  |  | Grade 1 NAFLD without advanced fibrosis | 38,216 | 865 | 2.47 | 1.008 (0.941, 1.080) |  |
|  |  | Grade 1 NAFLD with advanced fibrosis | 182,810 | 6,389 | 3.85 | 1.071 (1.038, 1.105) |  |
|  |  | Grade 2 NAFLD without advanced fibrosis | 16,273 | 405 | 2.72 | 1.104 (0.999, 1.221) |  |
|  |  | Grade 2 NAFLD with advanced fibrosis | 51,515 | 2,361 | 5.11 | 1.213 (1.155, 1.274) |  |
| Abdominal Obesity | No | No NAFLD | 2,005,820 | 30,075 | 1.64 | 1 (Ref.) | 0.006 |
|  |  | Grade 1 NAFLD without advanced fibrosis | 147,856 | 1,967 | 1.45 | 0.927 (0.884, 0.971) |  |
|  |  | Grade 1 NAFLD with advanced fibrosis | 319,103 | 7,710 | 2.66 | 1.063 (1.035, 1.092) |  |
|  |  | Grade 2 NAFLD without advanced fibrosis | 56,160 | 600 | 1.16 | 0.851 (0.784, 0.924) |  |
|  |  | Grade 2 NAFLD with advanced fibrosis | 58,881 | 1,451 | 2.74 | 1.186 (1.122, 1.252) |  |
|  | Yes | No NAFLD | 98,402 | 3,566 | 4.03 | 1 (Ref.) |  |
|  |  | Grade 1 NAFLD without advanced fibrosis | 56,810 | 1,369 | 2.64 | 0.918 (0.861, 0.977) |  |
|  |  | Grade 1 NAFLD with advanced fibrosis | 190,977 | 8,102 | 4.74 | 1.014 (0.974, 1.055) |  |
|  |  | Grade 2 NAFLD without advanced fibrosis | 106,942 | 1,916 | 1.96 | 0.951 (0.897, 1.009) |  |
|  |  | Grade 2 NAFLD with advanced fibrosis | 138,631 | 5,786 | 4.67 | 1.110 (1.061, 1.161) |  |
| Hypertension | No | No NAFLD | 1,741,579 | 18,640 | 1.16 | 1 (Ref.) | 0.026 |
|  |  | Grade 1 NAFLD without advanced fibrosis | 148,114 | 1,606 | 1.17 | 0.979 (0.929, 1.032) |  |
|  |  | Grade 1 NAFLD with advanced fibrosis | 317,058 | 6,062 | 2.09 | 1.122 (1.088, 1.158) |  |
|  |  | Grade 2 NAFLD without advanced fibrosis | 105,885 | 1,095 | 1.12 | 1.028 (0.964, 1.097) |  |
|  |  | Grade 2 NAFLD with advanced fibrosis | 98,456 | 2,085 | 2.33 | 1.259 (1.198, 1.322) |  |
|  | Yes | No NAFLD | 362,643 | 15,001 | 4.68 | 1 (Ref.) |  |
|  |  | Grade 1 NAFLD without advanced fibrosis | 56,552 | 1,730 | 3.38 | 0.933 (0.887, 0.982) |  |
|  |  | Grade 1 NAFLD with advanced fibrosis | 193,022 | 9,750 | 5.72 | 1.059 (1.030, 1.089) |  |
|  |  | Grade 2 NAFLD without advanced fibrosis | 57,217 | 1,421 | 2.73 | 0.981 (0.925, 1.039) |  |
|  |  | Grade 2 NAFLD with advanced fibrosis | 99,056 | 5,152 | 5.91 | 1.193 (1.150, 1.239) |  |

IR, incidence rate; HR, hazard ratio; PY, person years; NAFLD, nonalcoholic fatty liver disease

Multivariable model was adjusted for age, sex, income levels, body mass index, lifestyle factors (smoking status, alcohol consumption, physical activity), diabetes mellitus hypertension, dyslipidemia and chronic kidney disease.
